# Synthesis of MnO_2_–CuO–Fe_2_O_3_/CNTs catalysts: low-temperature SCR activity and formation mechanism

**DOI:** 10.3762/bjnano.10.85

**Published:** 2019-04-11

**Authors:** Yanbing Zhang, Lihua Liu, Yingzan Chen, Xianglong Cheng, Chengjian Song, Mingjie Ding, Haipeng Zhao

**Affiliations:** 1 College of Materials and Chemical Engineering, Henan University of Urban Construction, Pingdingshan 467000, People′s Republic of China; 2 Engineering Laboratory of Henan Province for Efficient Utilization of Coal Salt Resources, Pingdingshan 467000, People′s Republic of China

**Keywords:** amorphous materials, carbon nanotubes, low-dimensional materials, low-temperature catalysis, SCR activity

## Abstract

MnO_2_–CuO–Fe_2_O_3_/CNTs catalysts, as a low-dimensional material, were fabricated by a mild redox strategy and used in denitration reactions. A formation mechanism of the catalysts was proposed. NO conversions of 4% MnO_2_–CuO–Fe_2_O_3_/CNTs catalyst of 43.1–87.9% at 80–180 °C were achieved, which was ascribed to the generation of amorphous MnO_2_, CuO and Fe_2_O_3_, and a high surface-oxygen (O_s_) content.

## Introduction

Nitrogen oxides, NO*_x_* (*x* = 1, 2), contribute to acid rain, photochemical smog, greenhouse effect and ozone depletion [1–3]. The selective catalytic reduction of NO with NH_3_ (SCR), as a commercialized NO*_x_* abatement technology, has received a great deal of attention [4–5]. However, the catalyst of the SCR reaction, V_2_O_5_+WO_3_(MoO_3_)/TiO_2_, has some drawbacks, such as the toxic V**-**based material and the high operating temperature window (300–400 °C) [6–8]. Additionally, this kind of catalyst is easily influenced by ash and SO_2_, which makes it necessary to be installed downstream of electrostatic precipitator and desulfurizer, where the flue gas temperature is normally below 200 °C [9]. Therefore, it is of importance to develop a SCR catalyst with high catalytic activity below 200 °C.

Carbon nanotubes (CNTs), a low-dimensional material, exhibit a one**-**dimensional tubular structure and outstanding chemical and physical properties. Hence, they are extensively studied for the application in SCR, e.g., in MnO*_x_*/CNTs [10], Mn–CeO*_x_*/CNTs [11] and CuO*_x_*/carbonaceous-materials catalysts [12]. However, the working temperature window of these SCR catalysts is still between 200 and 300 °C.

A series of Cu-based [12–13] and (Mn + Fe)**-**based [14–15] catalysts have been applied in the SCR reaction and presented good catalytic activity. Nevertheless, the preparation procedures of the catalysts always need high**-**temperature calcination or high**-**pressure hydrothermal reactions, which are uneconomic and unsafe. Our previous studies, including MnO_2_–Fe_2_O_3_–CeO_2_–Ce_2_O_3_/CNTs [16] and Ce_2_O_3_–CeO_2_–CuO–MnO_2_/CNTs [17] catalysts, have reported a simple and mild redox method for the preparation of ternary and quaternary catalysts, and the resultant catalysts show outstanding denitration activity at 80–180 °C. The mechanisms of above preparation method are redox reactions between MnO_4_^−^ (from KMnO_4_) and Cl^−^ (from FeCl_3_ and CeCl_3_), or Mn^7+^ and O^2−^ (from KMnO_4_) as well as MnO_4_^−^ (from the KMnO_4_) and Cl^−^ (from CeCl_3_). The generation of Cl^−^ anions in the preparation process can result in corrosion of the equipment. On the basis of the above issues, a redox method with the formation of HNO_3_ between Mn^7+^ and O^2−^ (only from KMnO_4_) was developed, and the passivation through HNO_3_ can protect the metal equipment. This redox method yielded MnO_2_–CuO–Fe_2_O_3_/CNTs catalysts, and the as**-**synthesized catalysts were applied in the SCR reaction at 80–180 °C.

## Results and Discussion

### Catalytic activity

**Figure 1:**
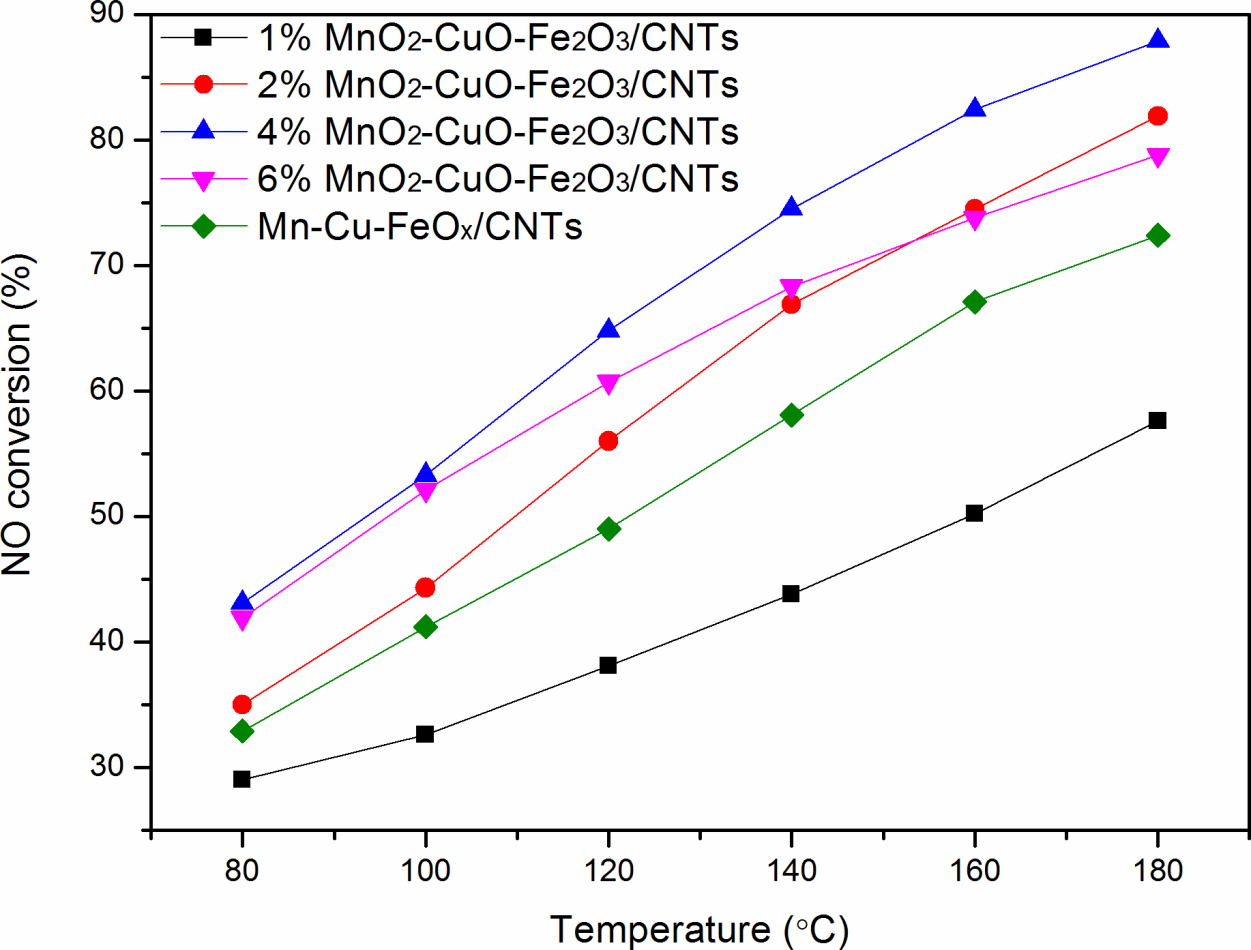
NO conversion as a function of the temperature over CNT**-**based catalysts. Reaction conditions: [NO] = [NH_3_] = 400 ppm, [O_2_] = 5%, N_2_ as balance gas, WHSV=280 L·g_cat_^−1^·h^−1^, 0.15 g catalyst.

Figure 1 shows the NO conversion as a function of temperature for the CNT**-**based catalysts. As shown in Figure 1, the NO conversion of MnO_2_–CuO–Fe_2_O_3_/CNTs prepared by the mild method was better than that of Mn–Cu–FeO*_x_*/CNTs**-**IWIM fabricated through incipient wetness impregnation, except for the 1% MnO_2_–CuO–Fe_2_O_3_/CNTs, and reached 69.9–87.9% between 140 to 180 °C. The SCR activity over 4% MnO_2_–CuO–Fe_2_O_3_/CNTs reached maximum values of 43.1–87.9% at 80–180 °C at a weight hourly space velocity of 280 L·g_cat_^−1^·h^−1^.

### X-ray diffraction measurements

**Figure 2:**
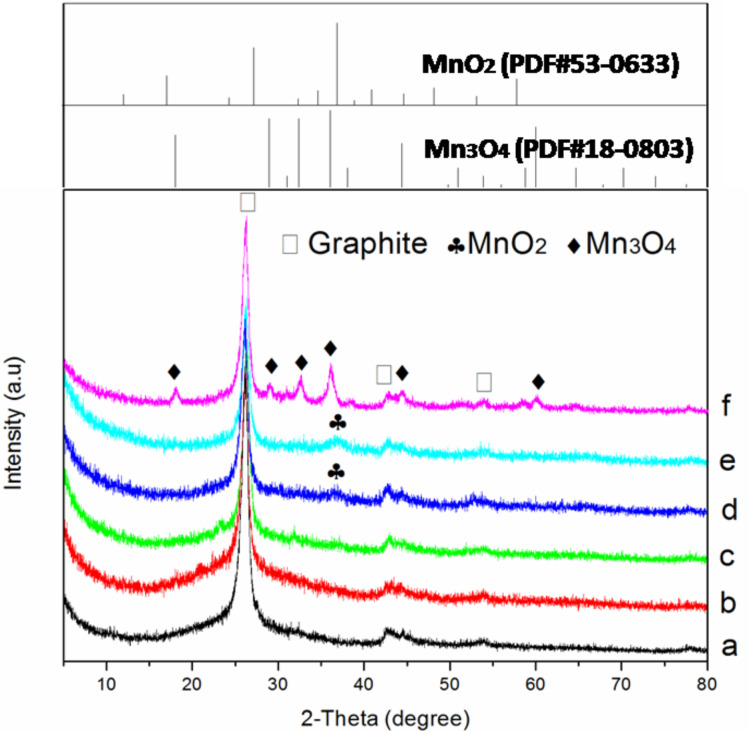
XRD patterns of the acid**-**treated CNTs and the as**-**synthesized catalysts: (a) acid**-**treated CNTs, (b) 1% MnO_2_–CuO–Fe_2_O_3_/CNTs, (c) 2% MnO_2_–CuO–Fe_2_O_3_/CNTs, (d) 4% MnO_2_–CuO–Fe_2_O_3_/CNTs, (e) 6% MnO_2_–CuO–Fe_2_O_3_/CNTs, and (f) Mn–Cu–FeO*_x_*/CNTs**-**IWIM.

Figure 2 shows the XRD patterns of the acid**-**treated CNTs and the as**-**synthesized catalysts. All samples present the characteristic diffraction peaks at 26.3°, 42.6° and 53.7°, corresponding to the (002), (100), and (004) planes of graphite, respectively [18]. For MnO_2_–CuO–Fe_2_O_3_/CNTs, only a weak peak of MnO_2_ (PDF#53**-**0633) can be observed when the loading was greater than or equal to 4%, whereas no diffraction peaks of metal oxides could be found, suggesting the formation of amorphous metal oxide phases. Amorphous catalytic materials are conducive to SCR activity [19], which is also shown in the results of NO conversion (Figure 1) and our previous studies [6,16–17]. In the case of Mn–Cu–FeO*_x_*/CNTs**-**IWIM, a series of peaks corresponding to Mn_3_O_4_ (PDF#18**-**0803) can be seen. Metal oxide catalysts with higher crystallinity show a smaller catalytic activity [20]. This is corroborated by the results of NO conversion. Besides, the intensities of the graphite peaks declines with increased loading, which is due to the interaction between the metal oxide catalysts and CNTs [21–25].

### Transmission electron microscopy and energy dispersive X-ray spectrometry

**Figure 3:**
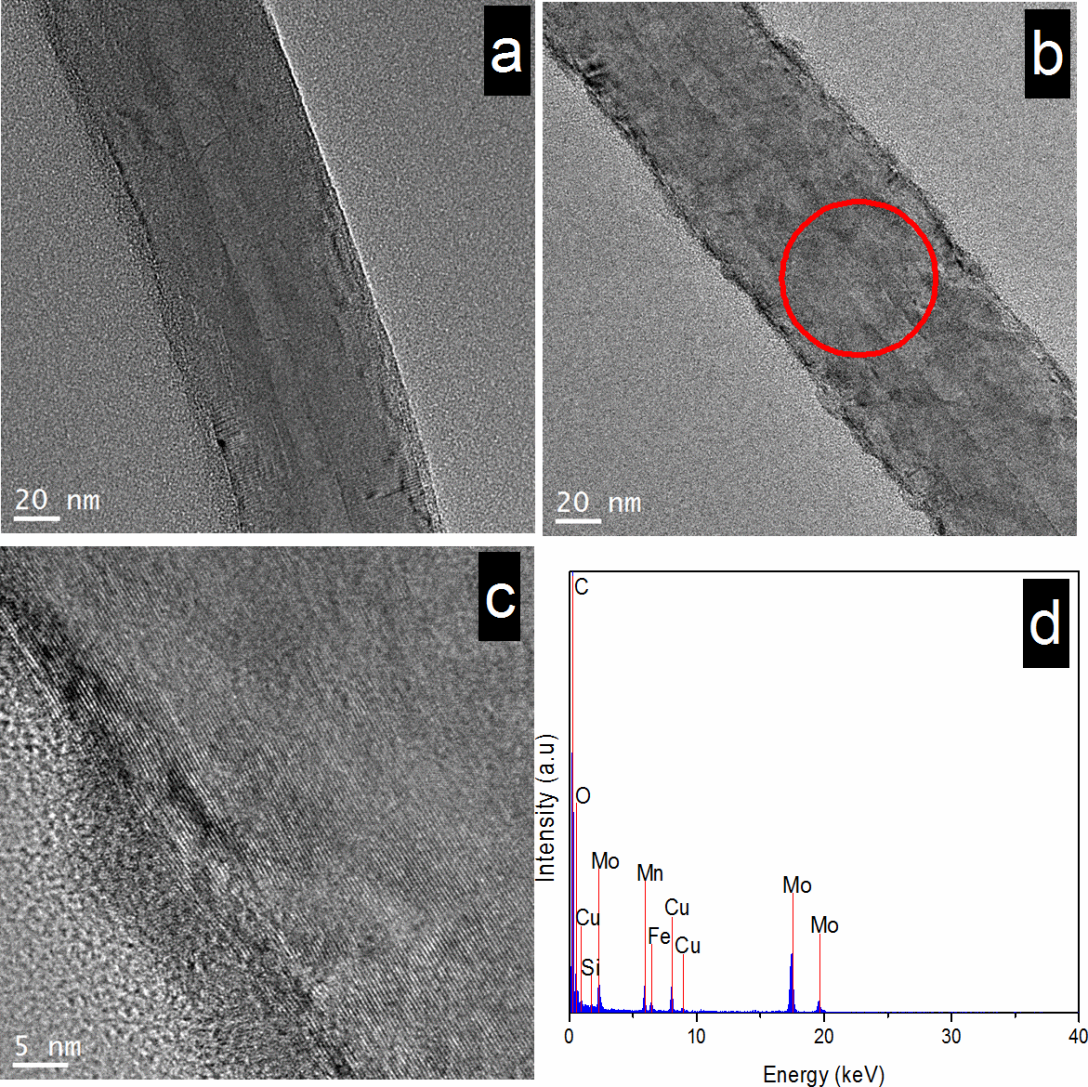
TEM and HRTEM images, as well as EDX spectrum of CNT**-**based samples: (a) CNTs, (b**–**d) 4% MnO_2_–CuO–Fe_2_O_3_/CNTs.

The morphologies of the acid**-**treated CNTs and the catalysts were investigated by TEM and HRTEM (Figure 3). The acid**-**treated CNTs have a smooth external surface (Figure 3a) that becomes coarse after being loaded with active metal oxide (Figure 3b). Additionally, the HRTEM images show the presence of catalysts nanoflakes, also verifying the generation of metal oxide catalysts on the CNT surface. The EDX spectrum (Figure 3d) shows signals of Mn, Cu, Fe, O and C. Clear lattice fringes of the metal oxides cannot be observed in the HRTEM images, indicating the generation of amorphous materials, which is consistent with the results of XRD (Figure 2).

### X-ray photoelectron spectroscopy

**Figure 4:**
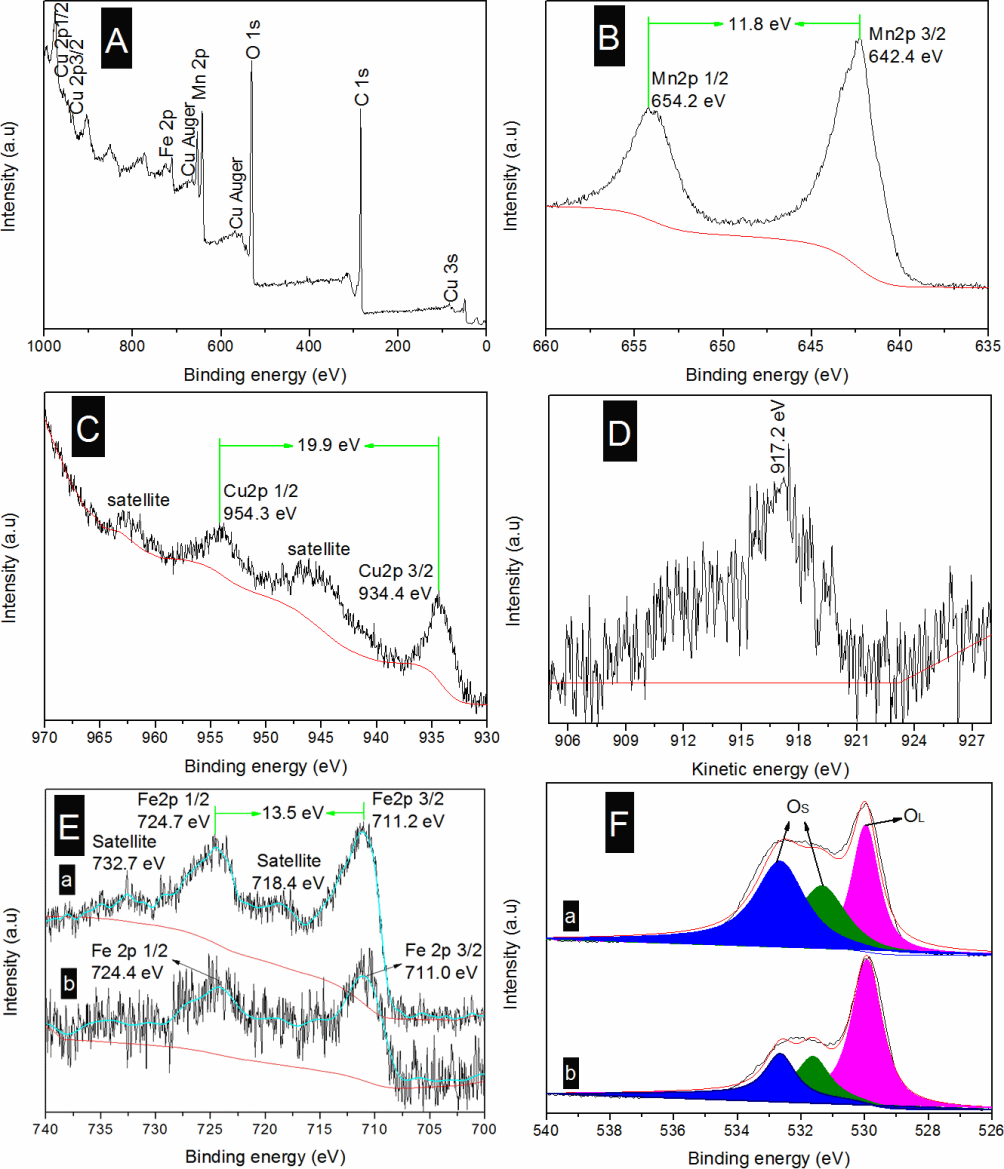
XPS results of the as**-**prepared catalysts: (A) XPS full**-**scan spectrum, (B) Mn 2p, (C) Cu 2p, and (D) Cu Auger spectra of 4% MnO_2_–CuO–Fe_2_O_3_/CNTs; (E) Fe 2p and (F) O 1s spectra for (a) 4% MnO_2_–CuO–Fe_2_O_3_/CNTs and (b) Mn–Cu–FeO*_x_*/CNTs**-**IWIM.

The XPS spectra of the as**-**prepared catalysts are given in Figure 4. The elements Mn, Cu, Fe, C, and O were detected in the XPS full-scan spectrum of Figure 4A. For the Mn 2p spectrum of 4% MnO_2_–CuO–Fe_2_O_3_/CNTs (Figure 4B), the binding energies at 654.2 and 642.4 eV, attributed to Mn 2p_1/2_ and Mn 2p_3/2_, respectively, can be observed. These values together with the energy separation of 11.8 eV demonstrate the formation of MnO_2_ [26]. The high oxidation state of MnO_2_ is advantageous to the SCR reaction [27], which is in accordance with the results of XRD and NO conversion measurements. The binding energies of Cu 2p_1/2_ and Cu 2p_3/2_ of the 4% MnO_2_–CuO–Fe_2_O_3_/CNTs catalyst (Figure 4C) are located at 954.3 and 934.4 eV, respectively, along with satellites at higher energies, indicating the formation of CuO [28]. The energy separation between Cu 2p_1/2_ and Cu 2p_3/2_ is 19.9 eV, also demonstrating the generation of CuO [29]. The Auger spectrum of Cu (Figure 4D) presents a peak at 917.2 eV, typical for CuO [30–31].

In the Fe 2p spectra of 4% MnO_2_–CuO–Fe_2_O_3_/CNTs and Mn–Cu–FeO*_x_*/CNTs**-**IWIM (Figure 4E, spectrum a), the Fe 2p_1/2_ and Fe 2p_3/2_ peaks at 724.7 and 711.2 eV, respectively, can be attributed to Fe_2_O_3_ [32]. The energy separation of 13.5 eV is typical for Fe_2_O_3_ [33]. The two satellites at 732.7 and 718.4 eV also verify the formation of Fe_2_O_3_ [34]. In spectrum b of Figure 4E, the binding energies of Fe 2p_1/2_ and Fe 2p_3/2_ (724.4 and 711.0 eV) of the Mn–Cu–FeO*_x_*/CNTs**-**IWIM catalyst appear at lower energies than those of 4% MnO_2_–CuO–Fe_2_O_3_/CNTs catalyst, revealing the formation of Fe_3_O_4_ [35]. Moreover, the absence of any satellites further proved the presence of Fe_3_O_4_. It is noteworthy that Fe_2_O_3_ exhibits a better low-temperature SCR activity than Fe_3_O_4_ [36], which is corroborated by the NO conversion measurements.

The O 1s peak can be divided into three peaks (Figure 4F). The peak at 529.9 eV is attributed to lattice oxygen (designated as O_L_), while the binding energies at 530.5**–**534.0 eV are ascribed to surface oxygen (labeled as O_S_). The O_S_ content (Table S2, Supporting Information File 1) of the 4% MnO_2_–CuO–Fe_2_O_3_/CNTs catalyst is 66.7%, whereas it is 36.8% in Mn–Cu–FeO*_x_*/CNTs**-**IWIM catalyst. O_S_ has a higher mobility than O_L_, which is in favor of the oxidation of NO to NO_2_, accelerating the SCR reaction [37]. This was also confirmed by the results of NO conversion and of previous studies [6,16–17].

### Scanning transmission electron microscopy

**Figure 5:**
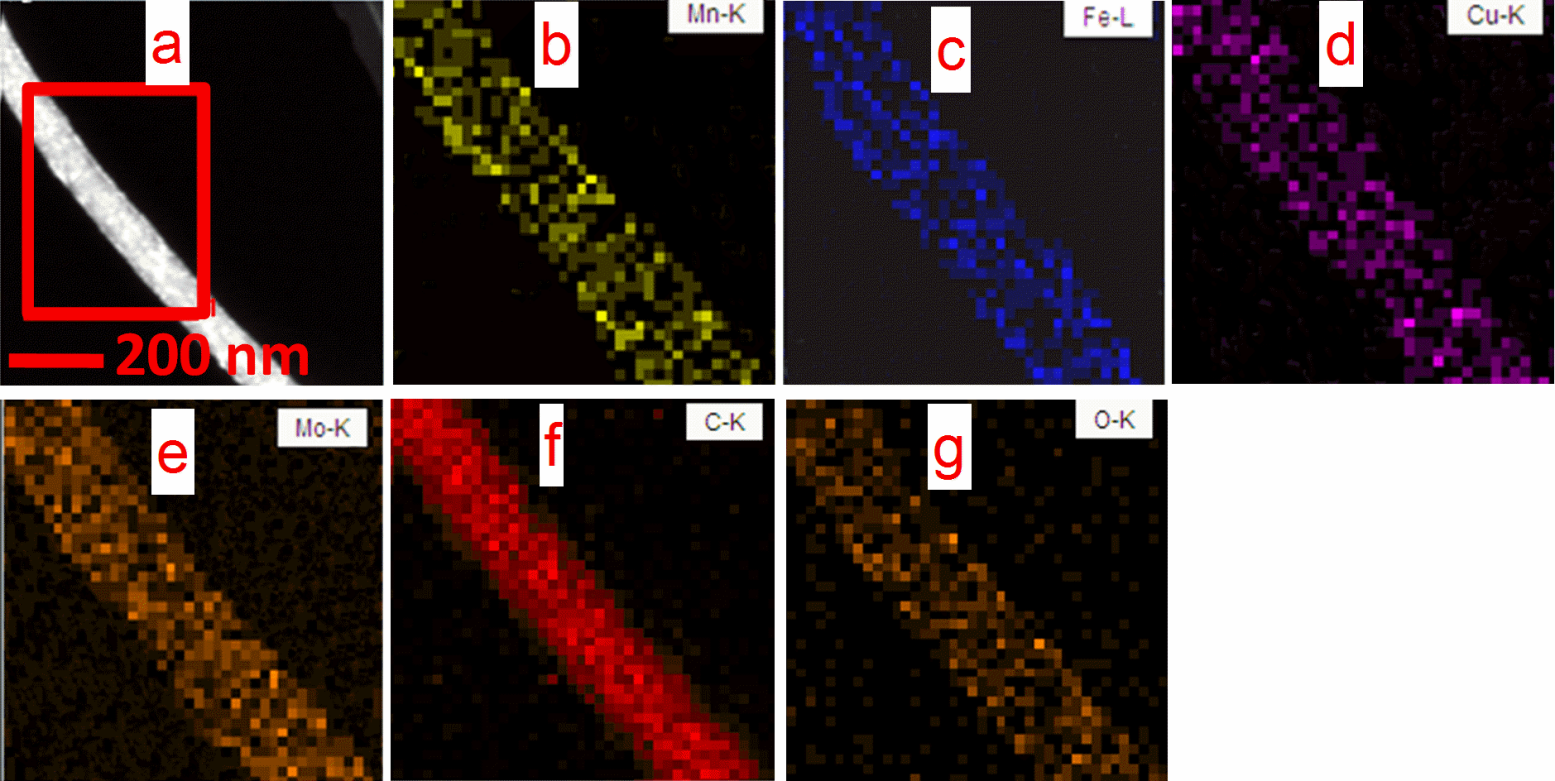
(a) STEM images and (b**–**g) element mappings of 4% MnO_2_–CuO–Fe_2_O_3_/CNTs.

STEM and element mapping were adopted to further investigate the morphology of the catalyst. As shown in Figure 5a, bright dots associated to the metal elements can be found, indicating the formation of metal oxide catalysts on CNTs. The STEM**-**EDX mappings (Figure 5b–g) exhibit a series of columnar element-distribution images, further proving that the metal oxide catalysts have been successfully loaded on the CNTs.

### Hydrogen temperature-programmed reduction analysis

**Figure 6:**
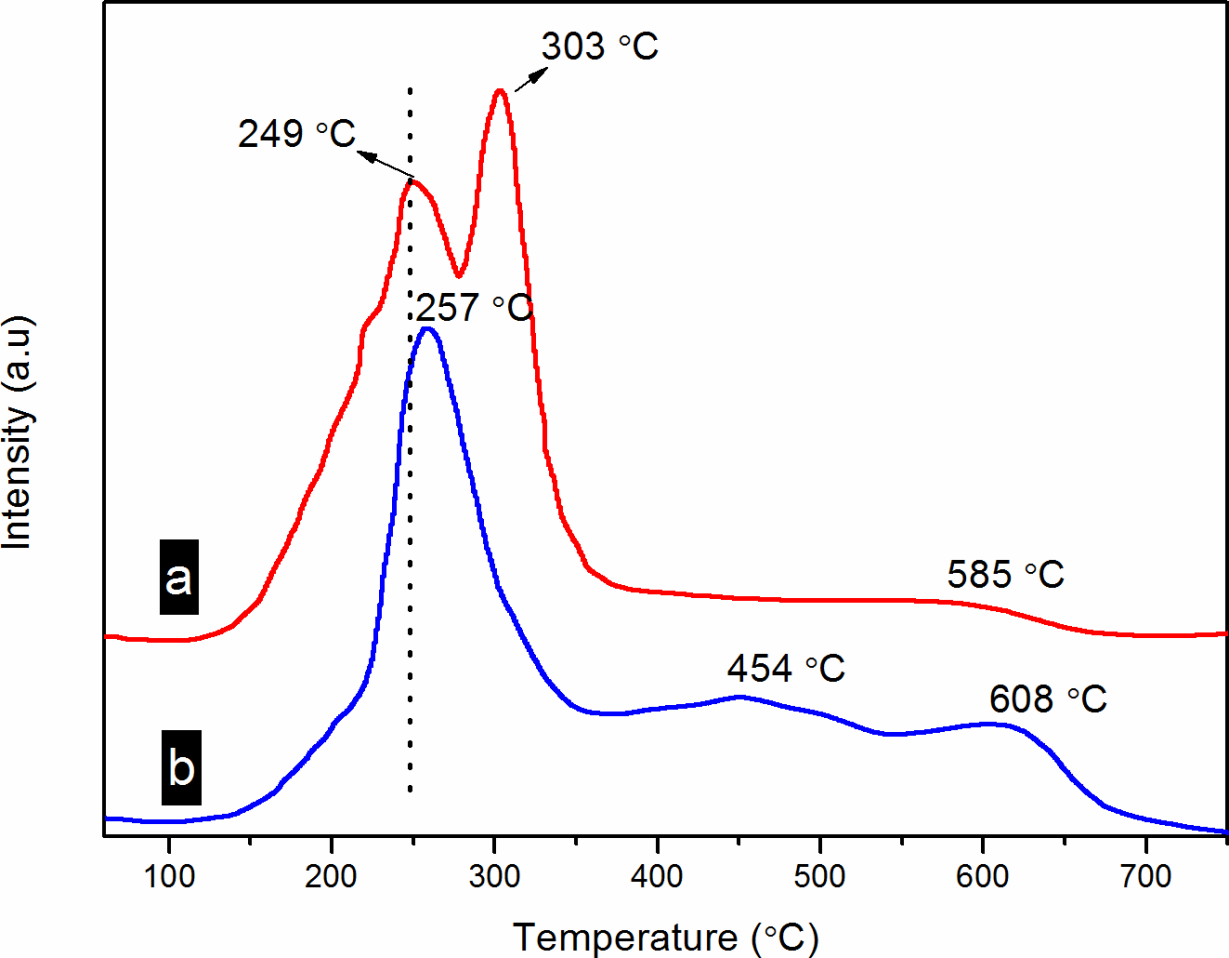
H_2_**-**TPR curves of two catalysts: (a) 4% MnO_2_–CuO–Fe_2_O_3_ /CNTs and (b) Mn–Cu–FeO*_x_*/CNTs**-**IWIM.

In SCR reaction, the redox performance of the catalyst plays a significant role in the catalytic cycle. Therefore, the reducibility of the as**-**obtained catalysts was evaluated by using hydrogen temperature-programmed reduction (H_2_**-**TPR). The results are listed in Figure 6. The catalysts exhibits three reduction peaks. For 4% MnO_2_–CuO–Fe_2_O_3_/CNTs catalyst (Figure 6a), the peaks at 150–300 °C (centered at 249 °C) are overlapping reduction peaks of MnO → Mn_2_O_3_ [26] and CuO → Cu_2_O [12,38–39]. The reduction peaks between 300–380 °C are overlapping peaks of Mn_2_O_3_ → Mn_3_O_4_ → MnO [26] and Cu_2_O → Cu [12]. Moreover, a reduction peak could be found at 580 °C, which is attributed to the oxygen groups on the CNT surface. For the Mn–Cu–FeO*_x_*/CNTs**-**IWIM catalyst (Figure 6b), the centers of the first and second reduction peaks (257 and 454 °C) were all at higher temperatures than those of 4% MnO_2_–CuO–Fe_2_O_3_/CNTs catalyst. This means that the reducibility is lower compared with that of 4% MnO_2_–CuO–Fe_2_O_3_/CNTs.

### Ammonia temperature-programmed desorption analysis

**Figure 7:**
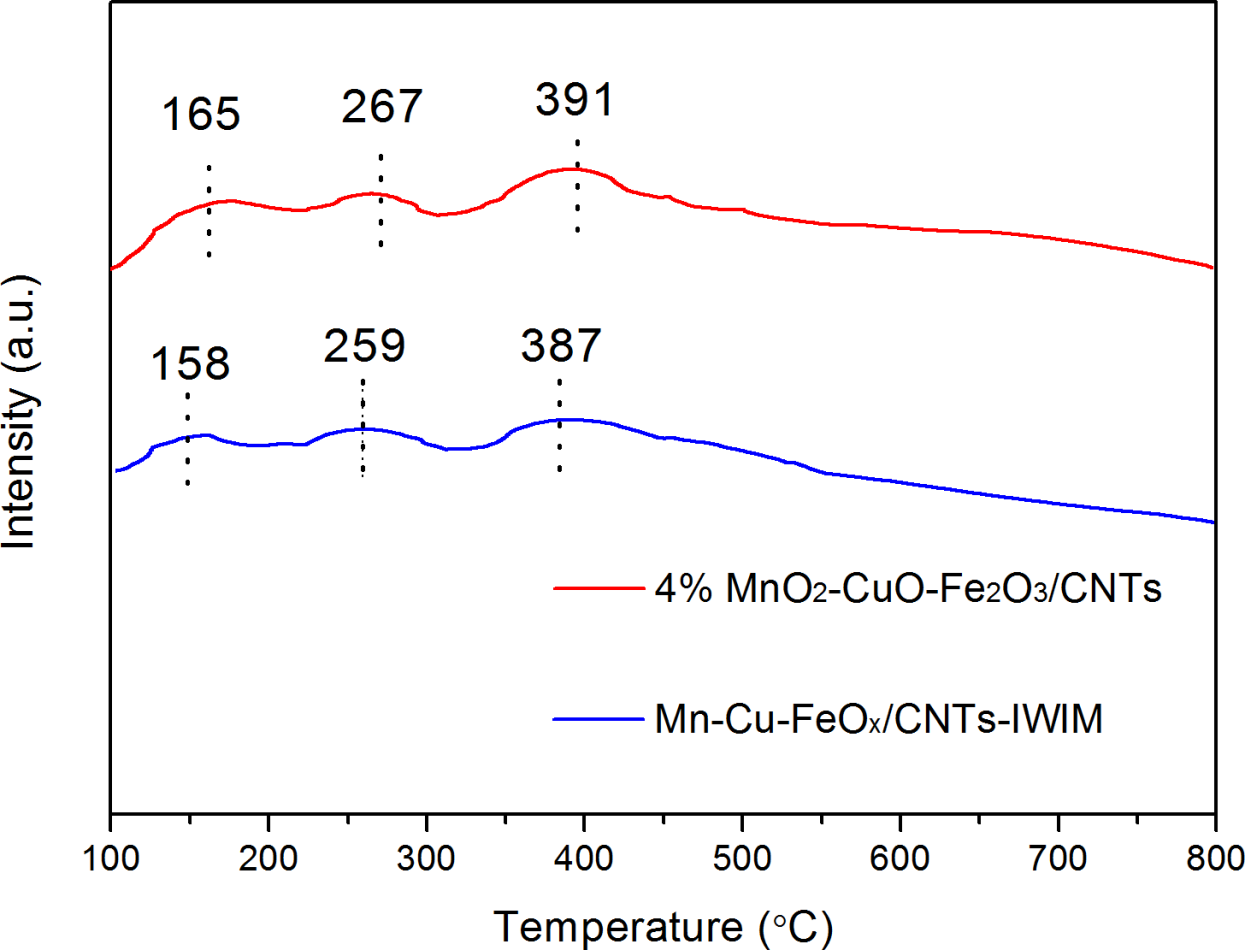
NH_3_-TPD curves of 4% MnO_2_–CuO–Fe_2_O_3_/CNTs and Mn–Cu–FeO*_x_*/CNTs-IWIM catalysts.

The chemisorption and activation of NH_3_ on the surface acid sites of a catalyst are generally viewed as the primary processes in the SCR of NO. Therefore, ammonia temperature-programmed desorption (NH_3_-TPD) measurements were carried out and the results are shown in Figure 7. The two catalysts presents three desorption peaks of NH_3_ corresponding to weak, intermediate and strong acid sites. For 4% MnO_2_–CuO–Fe_2_O_3_/CNTs catalyst, the centers of the three desorption peaks of NH_3_ are located at 165, 267 and 391 °C. These values are higher than those of Mn–Cu–FeO*_x_*/CNTs-IWIM catalyst (158, 259 and 387 °C), which means that the acid sites are stronger in 4% MnO_2_–CuO–Fe_2_O_3_/CNTs catalyst [11]. In addition, the number of Brønsted acid sites and Lewis acid sites at low and high temperature of the 4% MnO_2_–CuO–Fe_2_O_3_/CNTs catalyst is higher than that of the MnO_2_–CuO–Fe_2_O_3_/CNTs catalyst [40]. In general, stronger acid sites and a higher number of acid sites are advantageous to the SCR reaction [41], which is corroborated by the results of the NO conversion.

### Cyclic and long-term stability of catalysts

**Figure 8:**
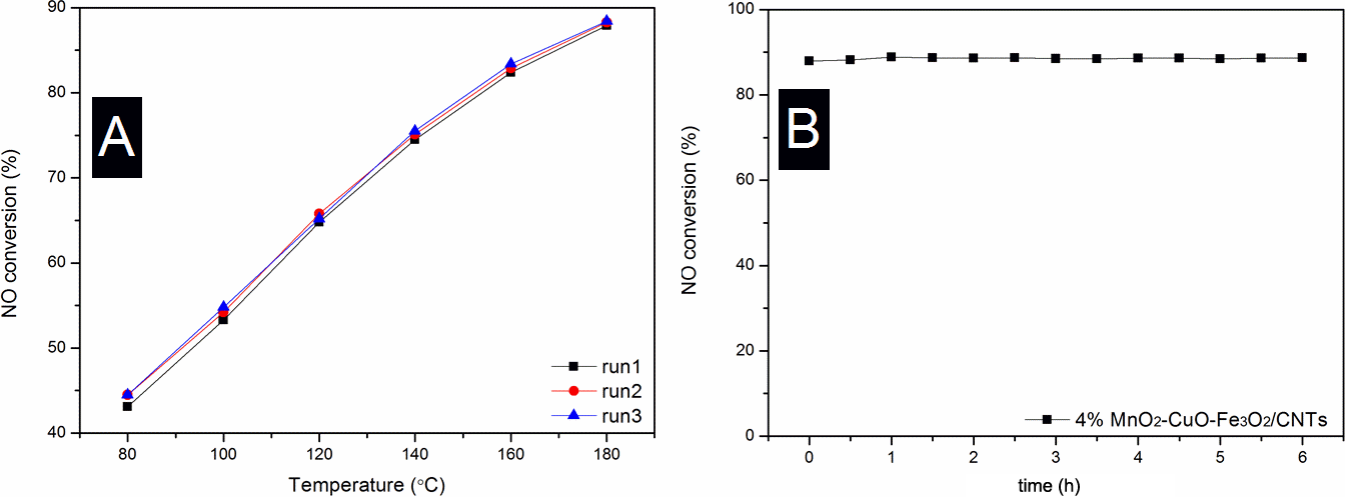
Cyclic and long-term stabilities of 4% MnO_2_–CuO–Fe_2_O_3_/CNTs catalyst.

In practical applications, the cyclic and long-term stability of a catalyst are crucial factors. The cyclic and long-term stability of the optimal 4% MnO_2_–CuO–Fe_2_O_3_/CNTs catalyst are listed in Figure 8. Figure 8A shows that the catalytic activity of 4% MnO_2_–CuO–Fe_2_O_3_/CNTs in run 2 and run 3 reaches 44.5–88.4% at 80–180 °C, which is similar with to the catalytic activity of 4% MnO_2_–CuO–Fe_2_O_3_/CNTs in run 1 (43.1–87.9%), suggesting an excellent cyclic stability of the catalyst. Figure 8B shows that the catalytic activity of 4% MnO_2_–CuO–Fe_2_O_3_/CNTs exhibits no obvious changes and reaches up to 87.9% at 180 °C during a test of 6 h, revealing the outstanding long-term stability. In view of the above favorable properties, the 4% MnO_2_–CuO–Fe_2_O_3_/CNTs catalyst will be potentially applicable in the low-temperature NO reduction with NH_3_.

### Comparison of the catalytic performance of three catalysts

Table 1 shows three catalysts with excellent performance in the low-temperature NO reduction with NH_3_, but the chemical reactions during preparation are different. The 6% Ce_2_O_3_–CeO_2_–CuO–MnO_2_/CNTs and 4% MnO_2_–Fe_2_O_3_–CeO_2_–Ce_2_O_3_/CNT catalysts present outstanding denitration efficiency values over the test temperature range, but Cl^−^ anions are formed in the preparation process, which might lead to a corrosion of metal equipment. The 4% MnO_2_–CuO–Fe_2_O_3_/CNTs catalyst reaches NO conversions of 43.1–87.9% at 80–180 °C, which is similar to two catalysts in our previous papers. Moreover, HNO_3_ is formed in the preparation process, which leads to an inactivation of the metal equipment.

**Table 1:** Catalytic performance of three catalysts.

catalyst	NO conversion at 80–180 °C and 180 °C (%)		weight hourly space velocity (L·g_cat_^−1^·h^−1^)

4% MnO_2_–CuO–Fe_2_O_3_/CNTs	43.1–87.9	87.9	280
6% Ce_2_O_3_–CeO_2_–CuO–MnO_2_/CNTs [17]	66.0–85.0	85.0	280
4% MnO_2_–Fe_2_O_3_–CeO_2_–Ce_2_O_3_/CNT [16]	52.8–99.4	99.4	210

### The generation mechanism for the MnO_2_–CuO–Fe_2_O_3_/CNTs catalyst

A reaction mechanism of the synthesis of the MnO_2_–CuO–Fe_2_O_3_/CNTs catalystis proposed. Based on the results of XRD and XPS, active components of MnO_2_, CuO, and Fe_2_O_3_ are formed. The following formation mechanism was inferred: Cu^2+^ and Fe^3+^ ions are first adsorbed on the surface of acid**-**treated CNTs via electrostatic interaction. Then the Cu(NO_3_)_2_ and Fe(NO_3_)_3_ are partly hydrolyzed in situ into Cu(OH)_2_, Fe(OH)_3_, and HNO_3_ on the CNTs. Afterwards, MnO_2_ is formed through the reaction between KMnO_4_ and HNO_3_, and the hydrolysis process is accelerated. MnO_2_–Cu(OH)_2_–Fe(OH)_3_/CNTs samples are obtained, and the MnO_2_–CuO_2_–Fe_2_O_3_/CNTs catalysts are prepared through thermal dehydration of the MnO_2_–Cu(OH)_2_–Fe(OH)_3_/CNTs samples [42–43]. The detailed reaction equations are:

[1]
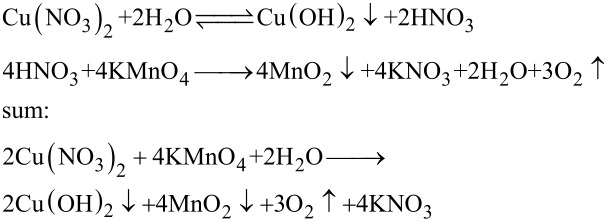


[2]
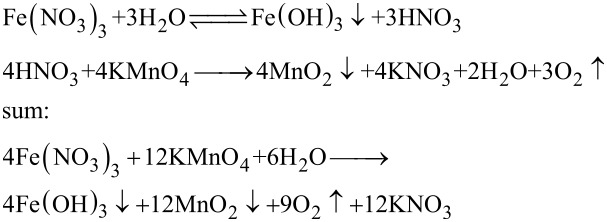


[3]
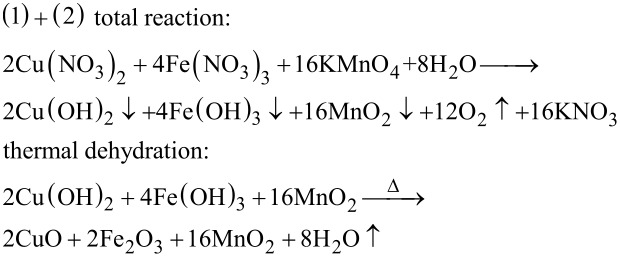


## Conclusion

MnO_2_–CuO–Fe_2_O_3_/CNTs catalysts were synthesized via a mild preparation method. The 4% MnO_2_–CuO–Fe_2_O_3_/CNTs catalyst showed the optimum low-temperature catalytic activity at 80–180 °C with a weight hourly space velocity of 280 L·g_cat_^−1^·h^−1^, benefitting from its amorphous metal oxide catalysts as well as high surface-oxygen content. The mild preparation conditions of the MnO_2_–CuO–Fe_2_O_3_/CNTs catalyst, can also provide a promising application in other catalytic fields.

## Experimental

### Materials

The raw CNTs (multi-wall) of 60–100 nm in diameter were purchased from Shenzhen Nanoport Company (China). KMnO_4_ (AR), Cu(NO_3_)_2_·3H_2_O (AR), Fe(NO_3_)·9H_2_O (AR) and ethanol (AR) were purchased from Shanghai Chemical Reagent Ltd. All chemical were used without further purification. Deionized water with a resistivity above 18.0 MΩ·cm was obtained from a JL-RO100 Millipore-Q Plus.

### Modification of CNTs and the synthesis of MnO_2_–CuO–Fe_2_O_3_/CNTs catalysts

The raw CNTs were first treated with HNO_3_ (65–68%) for 4 h at 140 °C, and then washed with deionized water and ethanol until pH 7. Finally, the solid product was dried at 70 °C for 12 h and grinded in an agate mortar.

First, acid-treated CNTs, Cu(NO_3_)_2_·3H_2_O, and Fe(NO_3_)·9H_2_O were dispersed in 40 mL deionized water under stirring for 12 h. Afterward, 40 mL of KMnO_4_ solution was added under continuous stirring at room temperature for another 12 h. Subsequently, the solid product was obtained by filtration, and washed with deionized water and ethanol until pH 7. Finally, the product was dried at 105 °C in air for 10 h. The as-prepared catalyst is denoted as *y* MnO_2_–CuO–Fe_2_O_3_/CNTs, where *y* represents the molar ratio of [KMnO_4_ + Cu(NO_3_)_2_ + Fe(NO_3_)_3_]/CNTs. For further details see [17]. The detailed molar ratios of precursors of 4% MnO_2_–CuO–Fe_2_O_3_/CNTs catalyst were obtained as follows: A molar ratio of 16 KMnO_4_:4Fe(NO_3_)_3_:2Cu(NO_3_)_2_ is obtained from Equations 1–3, and the molar ratio of [KMnO_4_ + Cu(NO_3_)_2_ + Fe(NO_3_)_3_]/CNTs = 4%. The mass of CNTs is 0.3 g (0.025 mol). The total amount of substance is then [KMnO_4_ + Cu(NO_3_)_2_ + Fe(NO_3_)_3_] = 4% × 0.025 mol = 0.001 mol. The amount of substance of KMnO_4_, Cu(NO_3_)_2_, and Fe(NO_3_)_3_ is 0.0007273 mol KMnO_4_, 0.0000909 mol Cu(NO_3_)_2_, and 0.0001818 mol Fe(NO_3_)_3_. For a comparative experiment, incipient wetness impregnation [44–45], as a common preparation method of catalysts, was applied to fabricate the Mn–Cu–FeO*_x_*/CNTs**-**IWIM catalyst with an optimal load of 4%.

### Characterization techniques

X**-**ray diffraction (XRD) was measured with an X'Pert Pro MPD X**-**ray diffractometer using Cu Kα radiation (λ = 0.15406 nm) with a 2θ range from 5° to 80°. Transmission electron microscopy (TEM) was performed on a JEOL model JEM 2010 EX instrument. Temperature**-**programmed reduction by H_2_ (H_2_**-**TPR) was assessed by using a custom-built TCD apparatus. Before the H_2_**-**TPR test, 50 mg catalyst was firstly purged in N_2_ at 200 °C for 1.5 h. The test was carried out in N_2_ (containing 6% H_2_) with a heating rate of 10 °C/min. X**-**ray photoelectron spectroscopy (XPS) was carried on a Thermo Scientific ESCALAB 250 spectrometer equipped with a dual Al/Mg anode (0.6 eV resolution).

### Catalytic activity

The SCR activity tests were carried out in a fixed**-**bed quartz reactor using 0.15 g catalyst in each test. The reaction gas consisted of [O_2_] = 5%, [NO] = [NH_3_] = 400 ppm, balanced by N_2_ gas. The total flow rate was 700 mL/min equivalent to a weight hourly space velocity (WHSV) of 280 L·g_cat_^−1^·h^–1^. A flue-gas analyzer (Kane International Limited, KM950) equipped with the NO, NO_2_, SO_2_, and O_2_ sensors was used to monitor the gas concentration. All data were recorded after 30 min till the catalytic reaction reached a steady state.

## Supporting Information

File 1 Additional experimental data.
