# Supplementary material for: Synthesis of MnO2–CuO–Fe2O3/CNTs catalysts: low-temperature SCR activity and formation mechanism
Source: Beilstein J Nanotechnol. 2019 Apr 11;10:848–55. doi: 10.3762/bjnano.10.85 (PMC6466767; doi:10.3762/bjnano.10.85)
Supplement: File 1 — Additional experimental data. [file Beilstein_J_Nanotechnol-10-848-s001.pdf]

BEILSTEIN JOURNAL OF NANOTECHNOLOGY

Supporting Information

for

Synthesis of MnO_2–CuO–Fe_2O_3/CNTs catalysts: low-temperature SCR activity and formation mechanism

Yanbing Zhang, Lihua Liu, Yingzan Chen, Xianglong Cheng, Chengjian Song, Mingjie Ding and Haipeng Zhao

Beilstein J. Nanotechnol. **2019,** *10,* 848–855. doi:10.3762/bjnano.10.85

Additional experimental data

License and Terms: This is a supporting information file under the terms of the Creative Commons Attribution License (http://creativecommons.org/licenses/by/4.0). Please note that the reuse, redistribution and reproduction in particular requires that the authors and source are credited.
The license is subject to the *Beilstein Journal of Nanotechnology* terms and conditions: (https://www.beilstein-journals.org/bjnano)

Table S1: BET surface area and pore volume for the pristine CNTs, acid-treated CNTs and as-prepared catalysts

sample	S_{BET} ($m^2 \cdot g^{-1}$)	pore volume ($cm^3 \cdot g^{-1}$)
pristine CNTs	63.11	0.1457
acid-treated CNTs	71.11	0.1585
1% Mn–CuO–Fe_2O_3/CNTs	61.29	0.1319
2% Mn–CuO–Fe_2O_3/CNTs	86.40	0.1660
4% Mn–CuO–Fe_2O_3/CNTs	95.66	0.1841
6%Mn–CuO–Fe_2O_3/CNTs	130.54	0.2447
Mn–Cu–FeO_x/CNTs-IWIM	111.05	0.1673

Table S2: Relative oxygen content of the as-prepared catalysts.

sample	O(%)	
	O_L	O_S
4% MnO_2–CuO–Fe_2O_3 /CNTs	33.3	66.7
Mn–Cu–FeO_x/CNTs-IWIM	63.2	36.8

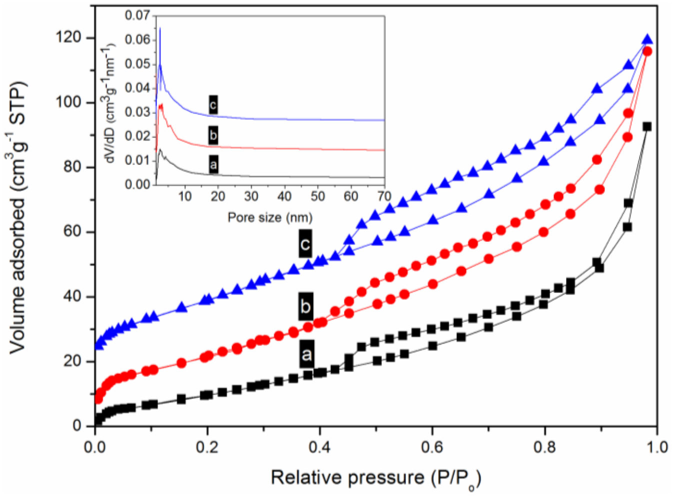

Figure S1: N_2 adsorption–desorption isotherm and pore size distribution (inset) of (a) acid-treated CNTs, (b) 4% MnO_2–CuO–Fe_2O_3/CNTs, and (c) Mn–Cu–FeO_x/CNTs-IWIM.

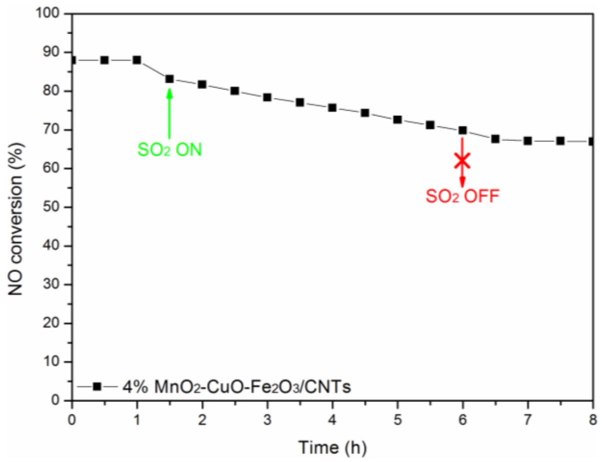

Figure S2: SO_2 tolerance of 4% MnO_2–CuO–Fe_2O_3/CNTs catalyst. Reaction conditions: [NO] = [NH_3] = 400 ppm, [SO_2] = 50 ppm (when used), [O_2] = 5%, N_2 as balance gas, WHSV=280 $L \cdot g_{cat}^{-1} \cdot h^{-1}$, 0.15 g catalyst.

BET surface area data

The results of BET surface area measurements (Table S1) show that the surface area of the pristine CNTs was 63.11 $m^2 \cdot g^{-1}$, whereas it became larger after being loaded with catalyst, indicating the even distribution of metal-oxide catalysts on the CNTs. It should be noted that although the 4% MnO_2–CuO–Fe_2O_3/CNTs catalyst showed the best catalytic activity among all samples, it possessed a smaller surface area (95.66 $m^2 \cdot g^{-1}$) than Mn–Cu–FeO_x/CNTs-IWIM (111.05 $m^2 \cdot g^{-1}$), revealing that the surface area is not the only important factor in SCR activity [1].

N_2 adsorption-desorption curves

Figure S1 shows the N_2 adsorption–desorption isotherm and the pore size distribution of the acid-treated CNTs and the as-synthesized catalysts. All samples present a typical type-IV isotherm along with a type-H4 loop, verifying the mesoporous structure [2,3]. The pore sizes of the samples were between 2.5 and 5.0 nm.

SO_2 tolerance

SO_2, a common component of flue gas, can inhibit the catalytic activity. Therefore, the catalysts need to be resistant against SO_2. In Figure S2, it is shown that the denitration efficiency of 4% MnO_2–CuO–Fe_2O_3/CNTs catalyst is ca. 87.9% in the absence of SO_2. When 50 ppm SO_2 is added to the gas flow for 5 h, there is a decline in catalytic efficiency to ca. 67.6%. Afterwards, the catalytic activity remains stable at around 67.1%. Hence, the 4% MnO_2–CuO–Fe_2O_3/CNTs exhibits SO_2 tolerance, which is favorable for its practical application.

References

1. Zhang, Y.; Zheng, Y.; Wang, X.; Lu, X. *Catal. Commun.* **2015,** *62,* 57–61. doi:10.1016/j.catcom.2014.12.023
2. Schill, L.; Putluru, S.; Fehrmann, R.; Jensen, A. *Catal. Lett.* **2014,** *144,* 395–402. doi:10.1007/s10562-013-1176-2
3. Sing, K.; Everet, D.; Haul, R.; Moscou, L.; Pierotti, R.; Rouquerol, J.; Siemieniewska, T. *Pure Appl. Chem.* **1985,** *57,* 603–619. doi:10.1351/pac198557040603
